# Pertussis outbreak investigation of Mekdela district, South Wollo zone, Amhara region, North-West Ethiopia

**DOI:** 10.1186/s13104-017-2735-1

**Published:** 2017-08-22

**Authors:** Sefi Derib Alamaw, Addisu Workineh Kassa, Yalemzewod Assefa Gelaw

**Affiliations:** 1 Institute of Public Health, College of Medicine and Health Sciences, University of Gondar, Gondar, Ethiopia; 2 Health Promotion and Disease Prevention Core Process, Amhara National Regional State Health Bureau, Bahir Dar, Ethiopia

**Keywords:** Pertussis, Outbreak, Mekdela, Northwest Ethiopia

## Abstract

**Background:**

Pertussis is a highly contagious respiratory illness caused by *Bordetella pertussis*. It is one of the most common vaccine-preventable bacterial infections that affects all susceptible individuals, regardless of age. Investigation was done to verify the existence of an outbreak and to identify associated risk factors contributed for the occurrence of an outbreak in Tork and Warkaye villages of Mekdela district.

**Methods:**

Unmatched community based case control and descriptive cross sectional investigation were conducted with one to two ratios. We used structured questionnaire to collect data from cases and controls.

**Results:**

A total of 215 cases and eight deaths were identified with an overall attack rate of 1.3 per 1000 population. The mean age of the cases was 3.7 years which was ranged from 3 months to 45 years. The more affected groups were females. On multivariate logistic regression analysis, the risk factor that remained independently statically significant associated with developing pertussis was presence of infected person in the family AOR (adjusted odds ratio): 5.859, (95% CI 2.526–13.589). But previously sick with pertussis AOR: 0.053, (95% CI 0.006–0.460) and receiving full dose of vaccine AOR: 0.256, (95% CI 0.080–0.818) were remained as protective factors from pertussis infection.

**Conclusions:**

The suspected pertussis outbreak was occurred in remote pocket villages/kebeles of Mekdela district. Routine immunization was not given regularly and functional refrigerators were not available in the health posts. Routine immunization services and treatment of infected patients with appropriate antibiotics should be intensified.

**Electronic supplementary material:**

The online version of this article (doi:10.1186/s13104-017-2735-1) contains supplementary material, which is available to authorized users.

## Background

Pertussis or whooping cough is a highly infectious respiratory illness caused by *Bordetella pertussis* or *Bordetella parapertussis* [1]. Pertussis has a wide distribution in many countries throughout the world. It is essentially a disease of infancy and early childhood, but at least half of the deaths resulting from pertussis infection occur in the first year of life. Although other agents like *B. parapertussis* and Adenovirus are associated with the etiology of whooping cough, at present the most important cause is *B. pertussis* [2]. Infants and young children have remained most susceptible to pertussis-related morbidity and mortality. In recent years infants younger than 6 months who are not old enough to have received three doses of the diphtheria–tetanus–pertussis vaccine and under vaccinated preschool children have been at higher risk for pertussis-associated complications [3].

WHO estimated that in 2008, about 16 million cases of pertussis occurred worldwide, 95% of which were in developing countries, and that some 195,000 patients died from this disease [4].

Clinical manifestation of pertussis, in the first stage, the catarrhal stage, is characterized by the insidious onset of coryza (runny nose), sneezing, low-grade fever, and a mild, occasional cough, similar to the common cold. The cough gradually becomes more severe, and after 1–2 weeks, the second, or paroxysmal stage, begins. Fever is generally minimal throughout the course of the illness. During paroxysmal stage, diagnosis of pertussis is usually suspected. Characteristically, the patient has bursts, or paroxysms, of numerous, rapid coughs, apparently due to difficulty expelling thick mucus from the tracheobronchial tree. At the end of the paroxysm, a long inspiratory effort is usually accompanied by a characteristic high-pitched whoop. Vomiting and exhaustion commonly follow the episode. Paroxysmal attacks occur more frequently at night, with an average of 15 attacks per 24 h. During the first 1 or 2 weeks of this stage, the attacks increase in frequency, remain at the same level for 2–3 weeks, and then gradually decrease. The paroxysmal stage usually lasts 1–6 weeks but may persist for up to 10 weeks [5]. Symptoms of pertussis is more severe in infants and young children. The clinical manifestations in adolescents and adults may be classical but are more often atypical.

The most common complication, and the cause of most pertussis-related deaths, is secondary bacterial pneumonia. Young infants are at highest risk for acquiring pertussis-associated complications. Since pertussis is a human disease; no animal or insect source or vector is known to exist. Adolescents and adults are an important reservoir for *B. pertussis* and are often the source of infection for children [6].

Pertussis is highly contagious. Patients are most infectious during the catarrhal period and the first 2 weeks after cough onset. It is contagious from symptom of onset to 21 or more days after the start of the paroxysmal cough or until completion of 5 days of appropriate antibiotic therapy [7].

In the 2012 whooping cough outbreak in the United Kingdom, the highest incidence was in infants and in babies under the age of 3 months [8]. Historically the introduction of pertussis vaccine resulted in a 92% decrease in morbidity and 93% decrease in mortality from whooping cough [9]. The aim of this investigation was to verify the existence of an outbreak and to identify associated risk factors contributed for the occurrence of the outbreak.

## Methods

### Study area and population

**Fig. 1:**
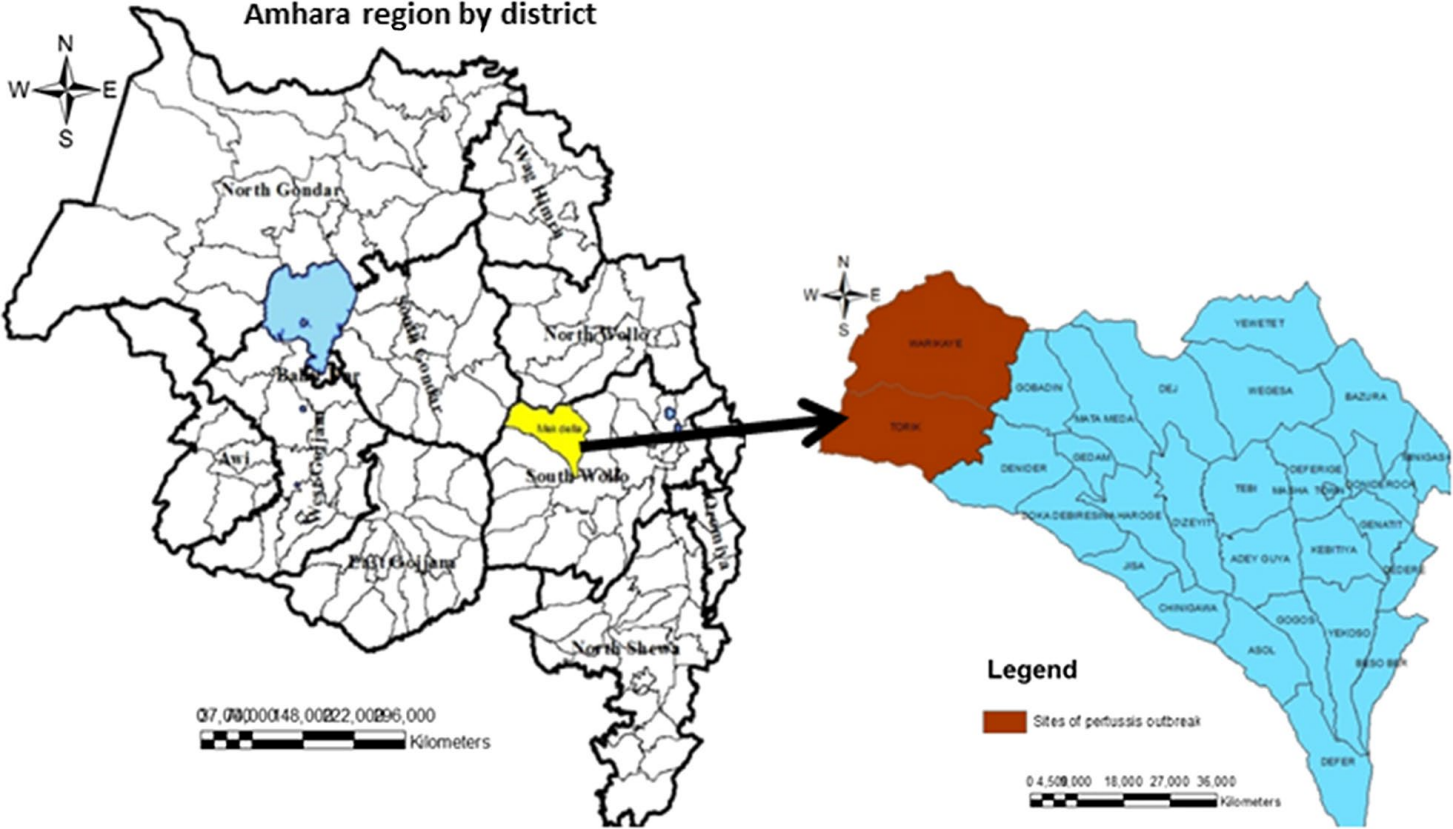
Map of Mekdela district, South Wollo zone, Amhara region, Ethiopia. The map is my own but the shape file source is central statistics of Ethiopia, 2007

Investigation was conducted in Tork and Warkaye villages/kebeles of Mekdela district. Mekdela is one of the districts of South Wollo zone of Amhara region. Mekdela is bordered by Legambo in the South, by Sayint in the West and in the East by Tenta districts of South Wollo zone and in the North by boarder by Simada district of South Gondar zone (Fig. 1).

### Case definitions

*Suspect case of pertussis* Non-improving cough of 14 days or more or cough of any duration with paroxysms or cough of any duration with whoop.

*Confirmed* A case of acute cough illness of any duration with a positive culture for *B. pertussis*, or a case that meets the clinical case definition and is confirmed by PCR, or a case that meets the clinical case definition and is epidemiologically linked directly to a lab-confirmed case.

*Epidemic of pertussis* is a situation when two or more cases clustered in time.

Mekdela district has 1 urban and 28 rural villages/kebeles. According to the 2007 national population and housing census, the total population of the district was estimated to be 166,185 in 2015. The district has seven health centers and 27 health posts but Warkaye village/kebele has no health post building and health extension worker. Health service coverage of the district is 100% by health center and the health post coverage is about 96.4% (was calculated from the number of health facilities divided by the projected population of Mekdela district). Tork and Warkaye are two of villages/kebeles that are found in Mekdela district which were affected by pertussis outbreak since 2015. These villages/kebele have a total population of 5190 each. Both villages were hard to reach pocket villages/kebeles of the district because there was no road construction.

### Study design

Unmatched community based case control and descriptive cross sectional investigation was conducted in fifty cases matched with one hundred controls that had no previous history of whooping cough living in the same village as the cases.

A suspected WHO working case definition was used to actively search for the cases in the communities and active case search was done at house to house level.

### Sample size determination and sampling

Fifty suspected cases and 100 controls were selected conveniently based on geographical accessibility.

### Data collection and procedure

Line list of cases was taken and followed daily within the study period. Cases and controls interviewed using structured questionnaire to collect demographic, possible factors; immunization status, etc. and house to house visits were also done to search active cases. All information hypothesized as risk factors for the pertussis outbreak were collected.

### Statistical methods

**Fig. 2:**
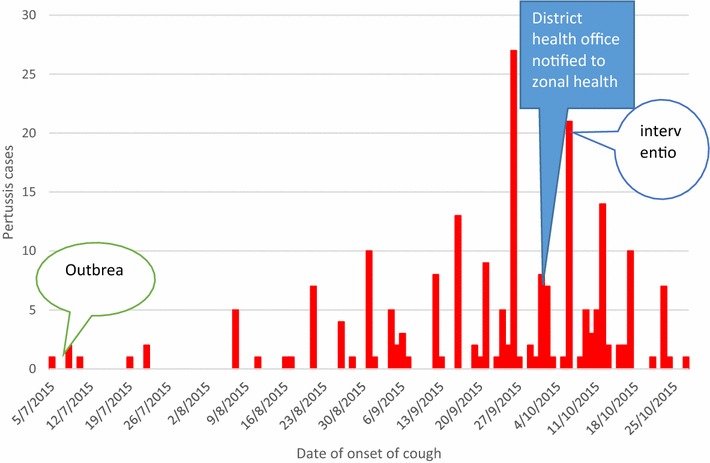
Epi curve of pertussis cases by date of onset of cough in Tork and Warkaye villages/kebeles, Mekdela district, South Wollo zone, Amhara, Ethiopia, from July 7th 2015 to October 25th 2015

Analysis was performed by Epi Info version 2007 and SPSS version 20 statistical software package. Frequency and percentage were calculated for the study variables. P value and two tail Fisher’s exact test was used to calculate and determine significance. In all statistical tests, the differences were considered to be statistically significant if P value less than 0.05 (Fig. 2).

## Result

In the descriptive study, during the outbreak period (July to October 2015) a total of 215 suspected pertussis cases with eight deaths were identified. From total eight deaths seven were from Tork village/kebele and one was from Warkaye village/kebele. In Tilma got/sub-village which is found in Tork village/kebele, six kids were died in three households, two kids in each household. Among the total cases 116 (54%) of them were females. In case control study, the age of the cases were ranged from 3 months to 45 years with mean age of 3.7 and median age of 3 years. Of the total cases, 123 (57.2%) of them were aged 0–4 years (Table 1).

**Table 1:** Distribution of pertussis cases by age group and sex in Tork and Warkaye kebeles/villages

Age group	Male	Female	Total number of cases (%)
0–4	59 (27.4%)	64 (29.8%)	123 (57.2)
5–9	28 (13%)	36 (16.7%)	64 (29.8)
10–14	8 (3.7%)	11 (5%)	19 (8.8)
≥15	4 (1.9%)	5 (2.3%)	9 (4.2)
Total	99 (46%)	116 (54%)	215 (100)

**Fig. 3:**
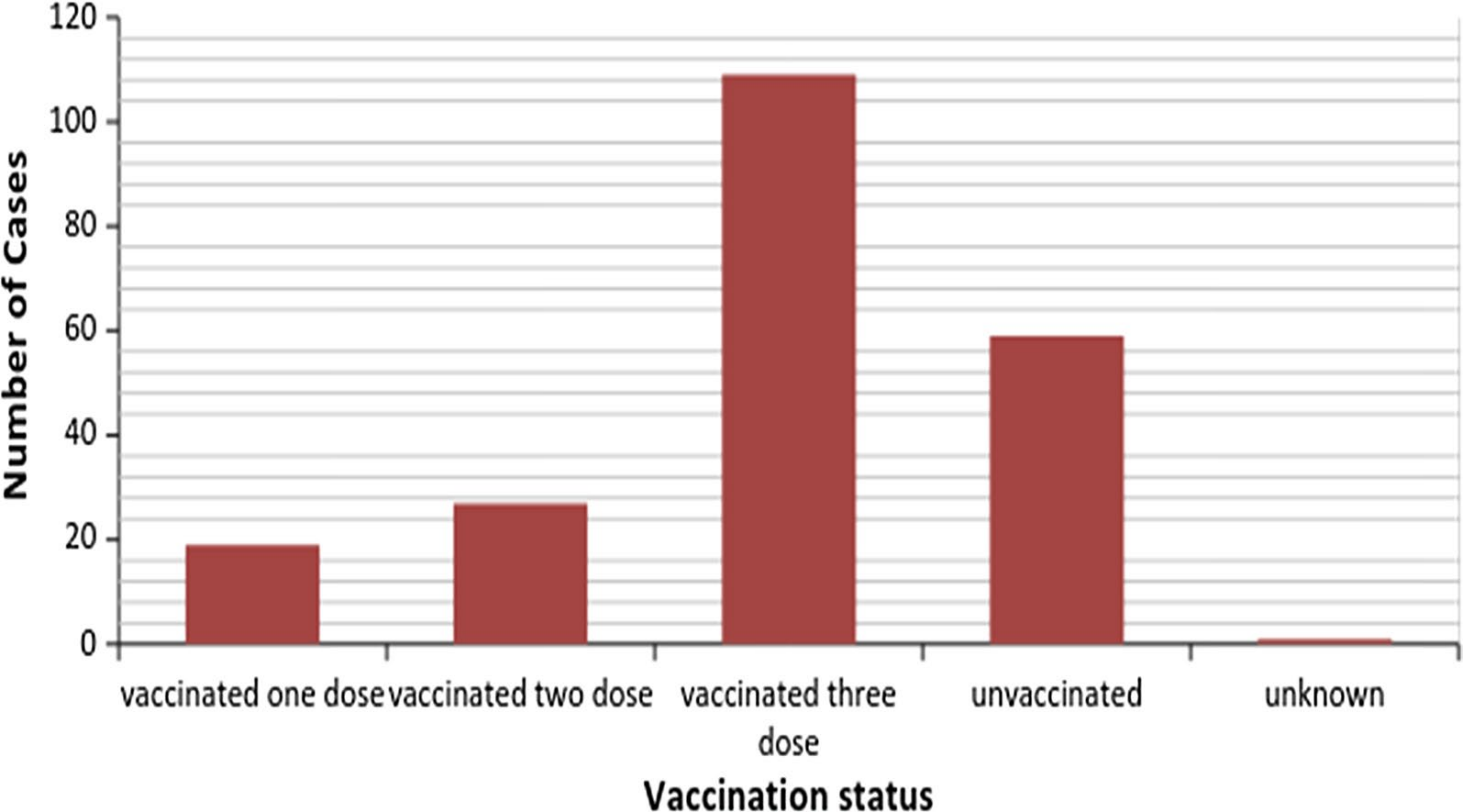
Pertussis vaccination status of pertussis cases in Tork and Warkaye kebele, Mekdela, South Wollo, Amhara, Ethiopia, 2015

In the health post EPI (Expanded Programme on Immunization) registration, the vaccination status of cases were 109 (50.7%), 27 (12.6%) and 19 (8.8%) were vaccinated for three dose, two dose and one dose respectively, while the remaining 59 (27.4%) and 1 (0.5%) were unvaccinated and unknown respectively (Fig. 3).

The overall attack rate of the disease was 1.3 per 1000 inhabitant of the two kebele and the case fatality rate (CFR) was 3.7 per 100 cases.

Majority of the cases were aged below 5 years old. The highest attack rate (6.8 per 1000) was among children of aged below 5 years. Individuals in the age group ≥15 years were the least affected with an attack rate of 0.09 per 1000 inhabitant of this age group. Highest case fatality rate, 6.3% was seen in children of age group 5–9 years. Majority of the deaths, five (62.5%) were occurred among females, with a case fatality rate of 4.3% (Table 2).

**Table 2:** Number of pertussis cases and deaths by age group and sex in Mekdela district, Amhara region

Variables	Total number of population	Number of cases	Number of deaths	AR/1000	CFR (%)
Age group in years					
0–4	18,166	123 (57.2%)	4 (50%)	6.8	3.3
5–9	23,432	64 (29.8%)	4 (50%)	2.7	6.3
10–14	23,378	19 (8.8%)	0 (0%)	0.8	0
≥15	101,209	9 (4.2%)	0 (0%)	0.09	0
Total	166,185	215 (100%)	8 (100%)	1.3	3.7
Sex					
Male	83,115	100	3	1.2	3
Female	83,070	115	5	1.4	4.34

### Vaccination coverage

Tork and Warkaye villages/kebeles health posts didn’t have functional refrigerators for the storage of vaccines, as result in these villages/kebeles there was no regular routine immunization service. The immunization service in this kebele was provided on an irregular period by transporting the vaccine from the Koreb health center. Immunization card was not given for vaccinated children. The health post report on pertussis showed that the vaccination coverage of Tork and Warkaye villages/kebeles were 92 and 100%, in the last year 2014/15 respectively. And also the district health office’s vaccination coverage was 100%.

### Analytical analysis

In this investigation a total of 50 cases and 100 controls who resided in the two villages/kebeles were selected for case control study, with a ratio of one case to two controls. Among the total 50 interviewed cases 26 (52%) of them were females and among the total 100 controls 53 (53%) of them were females. The age of the cases ranged from 3 months to 45 years with mean age of 3.7 years and median age of 3 years, whereas the age of the controls ranged from 11 months to 10 years with mean age of 4 years and median age of 5 years.

In bi-variate analysis; having contact with a person receiving one dose immunization to have pertussis during the last 2–3 weeks OR: 5.159 and contact receiving one dose immunization OR: 5.159 (95% CI 1.863–14.288) and presence of infected person in the family OR: 11.49 (1.49–88.5) were significantly associated with pertussis infection. But a person was sick previously and absence of exposure OR: 0.193 (0.089–0.417) were significantly associated with not developing pertussis infection.

In-multivariate analysis we identified one factor that remained independently associated with pertussis infection in Tork and Warkaye villages outbreak; presence of infected person in the family AOR = 5.86 (95% CI 2.53–13.59) (Table 3). In addition a person receive full dose of vaccine were found to be protective against pertussis infection.

**Table 3:** Multivariate analysis of factors of pertussis, Mekdela district, South Wollo zone, Amhara, Ethiopia

Variables	Case N = 50	Control N = 100	AOR (95% CI)	P value
Parent perception about root cause of pertussis				
Infected person in the family				
Yes	21	11	5.86 (2.5–13.6)	<0.001
No	29	89		
Became sick previously				
Yes	1	19	0.053 (0.006–0.46)	0.008
No	49	81		
Full dose				
Yes	5	27	0.256 (0.08–0.82)	0.021
No	45	73		

## Discussion

Over the period of outbreak (8 July 2015 to 27 October 2015) a total of 215 cases and eight deaths were line listed from the two villages/kebeles. The overall attack rate of the outbreak was 1.3 per 1000 inhabitants, which is less than pertussis outbreak in Papua New Guinea (4%) [9]. This might be due to under reporting of cases or weak surveillance activities.

More than half of pertussis cases (110) had received three doses of pertussis vaccine, this could be due to the vaccine might not be in good condition to prevent pertussis infection.

The case fatality rate of district was 3.7% which is higher as compared to the study done in Papua (3%), this could be due to poor accessibility of health service in the study area [9].

Females were more affected than males. The outbreak affected age ranges from 3 months to 45 years. The most affected age groups were those who were aged under 4 years. In this outbreak eight deaths were occurred of which five (62.5%) were females. This investigation identified the factor that the presence of infected person in the family 5.86 times that remained independently significantly associated with the occurrence of pertussis outbreak in Tork and Warkaye kebele of Mekdela district, South Wollo zone.

But we identified protective factors that are; children who received full dose of pertussis vaccine and he/she was sick previously, which showed that previously affected patients are rarely infected [4]. The likelihood contributing factor for the mortality of cases might be pertussis related complication, malnutrition and poor seeking behavior of people because of unreachable health facility, is far from their living environment.

The most affected age group was under 4 years 123 (57.2%) with the attack rate of 6.8 per 1000 population and four (50%) deaths with CFR of 3.3% occurred in children aged under 4 years which was higher than the CFR in West Africa (2.8%). This might be due to poor seeking behavior of the population. The highest case fatality (6.3%) was seen in children 5–9 years.

Even though vaccination coverage of the affected villages/kebeles was greater than 90%, outbreak was occurred, this might be due to double reporting and poor potency of the vaccine.

## Limitation of the study

Absence of vaccination card was difficult to determine the vaccination status, exact date of vaccination and other relevant information which could cause information bias. Recall bias on the date of onset by the cases and their mothers since the investigation was conducted lately after 215 cases and eight deaths were occurred.

## Conclusions

This outbreak was occurred in remote pocket villages/kebeles of Mekdela district with a weak surveillance system and delayed reporting. The outbreak was reporting after 3 months of the occurrence of deaths. In this outbreak the overall attack rate was 1.3 per 1000 population. More than half of the cases of this outbreak were children below 5 years of age. The factor that contributes for the occurrence of pertussis outbreak was presence of affected person in the family. The majority of cases and all deaths of the outbreak were occurred before the zonal health office, district health office and cluster health center being notified and initiated the response activities.

To prevent subsequent pertussis outbreaks the following action points put as recommendations:

- District health office should construct health posts to access routine health services for the community.
- Well functional refrigerators should be avail for the health posts to maintain the potency of vaccines.
- Rapid response team should be established at all levels to increase early notification of the outbreak.
- Inhabitants of the community needs to be mobilized to increase their awareness on importance of immunization and health service seeking behavior.
- The cluster health center should have to establish and implement routine EPI service in Tork and Warkaye villages/kebeles.
- The health extension workers in Tork health post and the health workers of Koreb health center in Warkaye village/kebele should enhance the awareness of the community on mode of transmission of pertussis, its prevention and importance of taking appropriate treatments if being infected to prevent pertussis related complications and death.

## Additional files

**Additional file 1.** Questionnaire-this questionnaire was our tool to collect all necessary information from the study participants.

**Additional file 2.** Line list-this is the list of all cases that we registered during out break investigation.

## Abbreviations

CFR: case fatality rate
EPI: Expanded Programme on Immunization
WHO: World Health Organization
