# Supplementary material for: Pertussis outbreak investigation of Mekdela district, South Wollo zone, Amhara region, North-West Ethiopia
Source: BMC Res Notes. 2017 Aug 22;10:420. doi: 10.1186/s13104-017-2735-1 (PMC5568300; doi:10.1186/s13104-017-2735-1)
Supplement: Supplementary file 1 — Additional file 1. Questionnaire-this questionnaire was our tool to collect all necessary information from the study participants. [file 13104_2017_2735_MOESM1_ESM.docx]

**Questionnaire for pertussis outbreak investigation**

Date_____

ID NO_________

Respondent: Patient Family member (Mother, Father: Brother or sister or other relatives close to patient.)

Investigators: ________________________, ________________________

Respondent’s status. 􀀀 Case 􀀀 Control

**A. socio demography**:

1. Age ________sex________

2. Residence: Woreda ____________, Kebele___________, Got___________

3. Occupation: ____________________________

4. Religion A. Orthodox B. Muslim

C. Protestant D. Others

5. Level of education

N/A KG

Primary Secondary

Tertiary Unable to read and write

6. Educational level of the mother/care giver

Not able to read and write

Primary

Secondary

Tertiary

7. Educational level of the father/care giver

Not able to read and write Primary

Secondary Tertiary Do not know

Total number of family members who live in the house -----------

Is there any person affected by the diseases in the family

1. Yes 2. No

**B. Clinical manifestations**

1. Do you have any of the following clinical features?

Paroxysmal cough Whoop

Post-tussive vomiting Apnea Cyanosis

Others Symptoms_____________________________________

2. Date of on set: ______________date/month/year

3. Date seen at health facility------/-----/------

**C. complications:**

Were any of the following present?

Edema of face 1. Yes 2. No

Sub Conjunctival hemorrhage 1. Yes 2. No

Weight loss 1. Yes 2. No

Pneumonia 1. Yes 2. No

Seizure 1. Yes 2. No

Hernia 1. Yes 2. No

Was participant hospitalized? 1. Yes 2. No

If yes, duration (in days) of hospitalization: ________________

**D. Possible source of exposure:**

1. Has the participant been exposed to a person(s) with a confirmed case of pertusis?

1. Yes 2. No **OR**

2. Has the participant been exposed to a person(s) with a cough lasting at least two (2) weeks with at least one of the following? Inspiratory “whooping”, post-tussive vomiting (vomiting immediately after coughing and without other apparent causes, paroxysms (fits of coughing)

1. Yes 2. No **OR**

3. Has the participant been exposed to a person with a possible case of pertusis (Case as ascertained by field worker based on mother/s, or other lay person/s, declaration)?

1. Yes 2. No

**If 1, 2 or 3 above is yes:**

Date of suspected contact: ____ / ____ / ____

What is the age of the contact case? ____ (yrs) ____ (mths) ____ (wks)

What was the date of onset in the contact case? ____ / ____ / ____

Relationship of contact case:–––––––––––––––––––

Had the contact case been adequately immunized (3 or more doses of pertusis vaccine)? 1. Yes 2. No 3. Unknown

**E. About Laboratory**

1. Is sample taken? Yes No

2. Date of collection of -------- sample: _______________

3. Date of specimen received by referral lab__________________

4. Did result reported? Yes No

5. If yes, is it positive? Negative Intermediate,

F.  **Treatment received:**

Was Erythromycin prescribed? 1. Yes 2. No

1. Prescribed for how many days? ________________

2. Was any other antibiotic prescribed? 1. Yes 2. No

If yes, specify? _____________________________________

Prescribed for how many days? ________________

**G. Immunization History**

1. Did he/she/you vaccinated against pertusis? No Yes

2. If yes; could you tell me the date of vaccination (see immunization card _________________

3. Number of dose received: _________________

4. If your answer is no for question 1 what is the main reason that you/your child not vaccinated

The health facility is far

I do not know the time of vaccination

The vaccine will hurt me /my child

The vaccine does not prevent pertusis other specify ---------------

5. Were/ was he/she/you ever been sick with such diseases? Yes No

6. If yes when? --------------------------

Do you know about mode transmission, prevention and control methods for pertusis?

1. YES 2. NO

**H. House condition**

Number of rooms

Does the house have windows? 1. YES 2. NO

If Yes, specify direction of windows ––––––––––––––
