# Supplementary material for: Pertussis outbreak investigation of Mekdela district, South Wollo zone, Amhara region, North-West Ethiopia
Source: BMC Res Notes. 2017 Aug 22;10:420. doi: 10.1186/s13104-017-2735-1 (PMC5568300; doi:10.1186/s13104-017-2735-1)
Supplement: Supplementary file 2 — Additional file 2. Line list-this is the list of all cases that we registered during out break investigation. [file 13104_2017_2735_MOESM2_ESM.docx]

Age ranges of Pertussis cases in the line list

1. <5=123
2. 5-14=83
3. ≥15=9

Total = 215
